# Oral probiotic combination of *Lactobacillus* and *Bifidobacterium* alters the gastrointestinal microbiota during antibiotic treatment for *Clostridium difficile* infection

**DOI:** 10.1371/journal.pone.0204253

**Published:** 2018-09-28

**Authors:** T. J. De Wolfe, S. Eggers, A. K. Barker, A. E. Kates, K. A. Dill-McFarland, G. Suen, N. Safdar

**Affiliations:** 1 Department of Biomedical Informatics, University of Pittsburgh, Pittsburgh, Pennsylvania, United States of America; 2 Department of Medicine, Division of Infectious Disease, University of Wisconsin – Madison School of Medicine and Public Health, Madison, Wisconsin, United States of America; 3 Department of Population Health Sciences, University of Wisconsin – Madison School of Medicine and Public Health, Madison, Wisconsin, United States of America; 4 William S. Middleton Memorial Veterans Hospital, Madison, Wisconsin, United States of America; 5 Department of Bacteriology, University of Wisconsin – Madison, Madison, Wisconsin, United States of America; Cleveland Clinic, UNITED STATES

## Abstract

Perturbations in the gastrointestinal microbiome caused by antibiotics are a major risk factor for *Clostridium difficile* infection (CDI). Probiotics are often recommended to mitigate CDI symptoms; however, there exists only limited evidence showing probiotic efficacy for CDI. Here, we examined changes to the GI microbiota in a study population where probiotic treatment was associated with significantly reduced duration of CDI diarrhea. Subjects being treated with standard of care antibiotics for a primary episode of CDI were randomized to probiotic treatment or placebo for 4 weeks. Probiotic treatment consisted of a daily multi-strain capsule (*Lactobacillus acidophilus* NCFM, ATCC 700396; *Lactobacillus paracasei* Lpc-37, ATCC SD5275; *Bifidobacterium lactis* Bi-07, ATCC SC5220; *Bifidobacterium lactis* B1-04, ATCC SD5219) containing 1.7 x 10^10^ CFUs. Stool was collected and analyzed using 16S rRNA sequencing. Microbiome analysis revealed apparent taxonomic differences between treatments and timepoints. Subjects administered probiotics had reduced Verrucomicrobiaceae at week 8 compared to controls. *Bacteroides* were significantly reduced between weeks 0 to 4 in probiotic treated subjects. *Ruminococcus* (family Lachnospiraceae), tended to be more abundant at week 8 than week 4 within the placebo group and at week 8 than week 0 within the probiotic group. Similar to these results, previous studies have associated these taxa with probiotic use and with mitigation of CDI symptoms. Compositional prediction of microbial community function revealed that subjects in the placebo group had microbiomes enriched with the iron complex transport system, while probiotic treated subjects had microbiomes enriched with the antibiotic transport system. Results indicate that probiotic use may impact the microbiome function in the face of a CDI; yet, more sensitive methods with higher resolution are warranted to better elucidate the roles associated with these changes. Continuing studies are needed to better understand probiotic effects on microbiome structure and function and the resulting impacts on CDI.

## Introduction

In the United States, *Clostridium difficile* has surpassed methicillin-resistant *Staphylococcus aureus* as the most common cause of hospital-acquired illness, responsible for over 450,000 infections and 29,000 deaths annually [1–3]. As a result, interventions to reduce the burden of *C*. *difficile* have become key priorities for federal funding agencies such as the Centers for Disease Control and Prevention, the United States Department of Veterans Affairs, and the Agency for Healthcare Research and Quality [4–6].

Antibiotic use is a major risk factor for *C*. *difficile* infection (CDI) as it induces ecological disruptions in the gastrointestinal (GI) microbiome thereby reducing the community’s ability to provide protective colonization resistance [7]. Many strains of *C*. *difficile* are resistant to antibiotics and have versatile genomes, allowing the acquisition of additional resistance genes and the ability to opportunistically proliferate in the host GI tract during antibiotic treatment [8]. Vancomycin, metronidazole, or fidaxomicin treatment is the standard of care for CDI; however, approximately 20% of patients have recurrent GI symptoms after primary treatment [3,9,10].

Supplemental probiotics are often recommended as microbiota-targeted therapies to improve CDI symptoms, but evidence for their efficacy is limited [11]. To further examine the evidence for probiotics in ameliorating symptoms associated with CDI, Barker et al. conducted the Probiotics for *C*. *difficile* infection in adults (PICO) study, a randomized, double-blinded, placebo-controlled clinical trial of health outcomes among subjects randomized to a multi-strain probiotic in addition to standard antibiotic treatment during a CDI episode [12]. In this trial, it was observed that subjects in the probiotic treatment group had significantly reduced duration of CDI diarrhea based on subject documented stool consistency data. For probiotics to be considered default CDI therapies, understanding the associated impacts to the GI microbiota is critical. Here, with stool samples from subjects enrolled in the PICO study, we sought to identify compositional changes to the GI microbiota associated with the supplemental probiotic treatment. Using the resulting compositional profiles, we then inferred the associated microbial community functions.

## Materials and methods

### Intervention

The PICO study was conducted between February 2013 and February 2015 with approval obtained from the Health Sciences Institutional Review Board at the University of Wisconsin—Madison (trial registered at clinicaltrials.gov, NCT01680874). The study sample size was originally determined in Barker et al. to test for the primary clinical outcome of reduced days diarrhea experienced by PICO subjects with a minimal detectable difference of two days [12]. Using a two-sided non-parametric Wilcoxon rank-sum test at an alpha significance level of 0.05, a total of 23 subjects per treatment arm were needed to achieve 80% power of detection. After adjusting for a 20% study dropout, 29 subjects in each study arm were necessary (for details including subject selection, recruitment, and demographics, see Barker et al. 2015 and Barker et al. 2017). After the enrollment period, 33 subjects experiencing a first episode of mild to moderate CDI were randomly assigned an oral placebo or multi-strain probiotic capsule (*Lactobacillus acidophilus* NCFM, ATCC 700396; *Lactobacillus paracasei* Lpc-37, ATCC SD5275; *Bifidobacterium lactis* Bi-07, ATCC SC5220; *Bifidobacterium lactis* B1-04, ATCC SD5219) containing 1.7 x 10^10^ CFU/capsule daily for four weeks (week 0 to week 4). The placebo capsule was identical to the probiotic in appearance and taste and contained an inert filler that was also contained in the probiotic capsule. At week 0 (prior to the first treatment dose), 4 (after treatment cessation), and 8 (after 4 weeks of subject follow-up), participants were directed to submit a stool sample that was immediately stored at -80 °C for further processing.

### DNA extraction

Approximately 200 mg of each fecal sample was added to individual 2 mL tubes containing 250 mg of 0.1 mm zirconia/silica beads, and a single 4.8 mm stainless steel bead. 210 μl of 20% sodium dodecyl sulfate and 500 μl of phenol:chloroform:isoamyl alcohol (25:24:1, pH 7.9) were added to each tube and vortexed. The tubes were subsequently lysed by mechanical disruption with a bead beater for 3 minutes at room temperature. Next, the tubes were centrifuged at 16,000 x *g* (4 °C) for 3 minutes for phase separation. The aqueous layer was transferred to individual 1.5 mL micro-centrifuge tubes. Next, 50 μl of 3 M sodium acetate and then 500 μl isopropanol were added to each micro-centrifuge tube. Tubes were inverted several times and incubated at −20 °C for 1 hour. Following incubation, tubes were centrifuged at 16,000 x *g* (4 °C) for 20 minutes to collect the DNA pellets, which were washed with 200 μl ethanol and air-dried. DNA pellets were suspended in 100 μl Tris-EDTA (TE) buffer overnight at 4 °C before being purified using a NucleoSpin Gel and PCR Clean-up kit (MACHEREY-NAGEL GmbH & Co. KG, Düren, Germany), according to the manufacturer’s directions, and eluted in 30 μl TE buffer. DNA was stored at -20 °C until further processing.

### Bacterial amplification and sequencing

PCR was performed using universal primers flanking the V4 region of the bacterial 16S rRNA gene [13]. A total of 25 ng DNA, 0.4 μM each primer, 12.5 μl 2X KAPA HiFi HotStart ReadyMix (Kapa Biosystems, Wilmington, US), and water to 25 μl were used for one reaction per sample. Cycling conditions began with an initial denaturation of 95°C for 3 minutes followed by 25 cycles of 95 °C for 30 seconds, 55 °C for 30 seconds, and 72 °C for 30 seconds, with a final extension at 72 °C for 5 minutes. PCR products were purified by gel extraction from a 1.0% low-melt agarose gel using the ZR-96 Zymoclean Gel DNA Recovery Kit (Zymo Research Corp, Irvine, US). Samples were quantified using a Qubit Fluorometer 2.0 (Invitrogen, Waltham, US) and pooled at equimolar concentrations. The pool plus 5% PhiX control DNA (Illumina, Inc, San Diego, US) was sequenced with the MiSeq 2x250 v2 reagent kit (Illumina, Inc, San Diego, US) using custom sequencing primers [13].

### Sequence analysis and statistics

All sequences were demultiplexed on the Illumina MiSeq and processed using mothur v1.38.1 [13,14]. Briefly, paired-end sequences were combined into contigs, and those of poor quality were removed. High-quality contigs were aligned against the full-length SILVA 16S rRNA gene reference alignment v123 and screened for alignment to the correct region [15]. Pre-clustering and chimera removal were performed to reduce sequencing and PCR error. Singletons were removed to facilitate downstream analyses. Sequences were then grouped into 97% operational taxonomic units (OTUs) by uncorrected pairwise distances and *de novo* average neighbor clustering. OTUs were classified using the Greengenes database v13.8.99 with the Wang method and a bootstrap cutoff of 80 [16,17]. Good’s coverage was calculated in mothur and the dataset was rarefied to 10,000 sequences per sample for all further analyses. All resulting DNA sequences have been deposited in the National Center for Biotechnology Information Sequence Read Archive (SRA: SRP101347).

We assessed α-diversity by the Shannon diversity index calculated in mothur [13,14]. Differences in community diversity were assessed overall by two-way repeated measure analysis of variance (ANOVA) after visual inspection of the quantile-quantile plot revealed Shannon to be roughly normal. Diversity was assessed for week, treatment, and week:treatment with the interaction term removed if not significant. Pairwise comparisons between treatments within time points and between time points within treatments were calculated using t-tests with the Benjamini-Hochberg correction for multiple comparisons in R v3.3.2 [18].

Beta-diversity distances measuring total community structure (relative abundance, Bray-Curtis) and composition (presence and absence, Jaccard) were calculated from square root transformed data in R using vegan v2.4–1 [19]. Distances were evaluated using nonmetric multidimensional scaling plots for differences across week, treatment, and week:treatment using permutational analysis of variance (PERMANOVA). Models were stratified by subject to account for repeated measures across time and the interaction term was removed if not significant. Pairwise comparisons between treatments within time points and between time points within treatments were performed with PERMANOVA with a Benjamini-Hochberg correction for multiple comparisons.

The genera, families, and OTUs contributing to differences seen in PERMANOVA were identified by analysis of similarity percentages (SIMPER) in vegan. The OTUs and genera contributing at least 2% of the variation and families contributing at least 5% in at least one pairwise comparison were considered relevant. The relevant taxa were subsequently assessed by non-parametric repeated measure ANOVA-type statistic (ATS) with the denominator degrees of freedom set to infinity using the nparLD v2.1 package[20]. Taxa that were significant prior to multiple comparison correction were assessed pairwise by non-parametric Dunn's tests. Both ATS and Dunn’s were then corrected for multiple comparisons using Benjamini-Hochberg. All tests were assessed at significance *p* ≤ 0.05 and trends 0.05 < *p* < 0.10.

### Functional prediction

To predict the microbial community function associated with probiotic administration at week 4, we normalized the 16S rRNA marker gene OTU table by copy number abundance in PICRUSt v1.1.1, then multiplied each by the Kyoto Encyclopedia of Genes and Genomes (KEGG) ortholog abundances predicted for each taxon [21–24]. The relative abundance of KEGG modules was calculated using HUMAnN2 v0.11.1 and differences in abundance were identified using linear discriminant analysis (LDA) effect size with LEfSe Galaxy v1.0 [25,26]. KEGG module differences were considered biologically significant above an LDA score of 2.0 and alpha value for the factorial Kruskal-Wallis test among treatments of 0.05.

## Results

Of the 31 subjects from the PICO study who submitted stool samples, 22 submitted a complete set containing samples from week 0, 4, and 8 (placebo, n = 11; probiotic, n = 11), six subjects submitted two samples from either weeks 0 and 4 (probiotic, n = 2) or weeks 4 and 8 (placebo, n = 2; probiotic, n = 2), and three submitted a single sample from week 0 (placebo, n = 1; probiotic, n = 1) or week 4 (placebo, n = 1) (Table 1). All stool samples acquired from the nine subjects who submitted incomplete sets were still included in the sequencing run. Sequencing of all samples yielded 4.56 million raw and 3.5 million clean sequences (mean 42,778 ± 25,478 s.d. per sample) after filtering in mothur. Sufficient sequencing was completed as all samples had a Good’s coverage > 99.5%.

**Table 1:** Quantity of samples collected during the PICO study.

	Week			Total
	0	4	8	
Placebo	12	14	13	39
Probiotic	14	15	13	42

Using the Bray-Curtis dissimilarity index, we quantified the differences in overall community structure. Samples were found to differ significantly by treatment (*p* = 0.001) and across time points (*p* = 0.001). Pairwise comparison and false discovery rate (FDR) correction of β-diversity between time points within discrete treatment groups and between treatments within discrete time points did not reveal significant treatment and time point comparisons that were meaningful (S1 Table).

**Fig 1:**
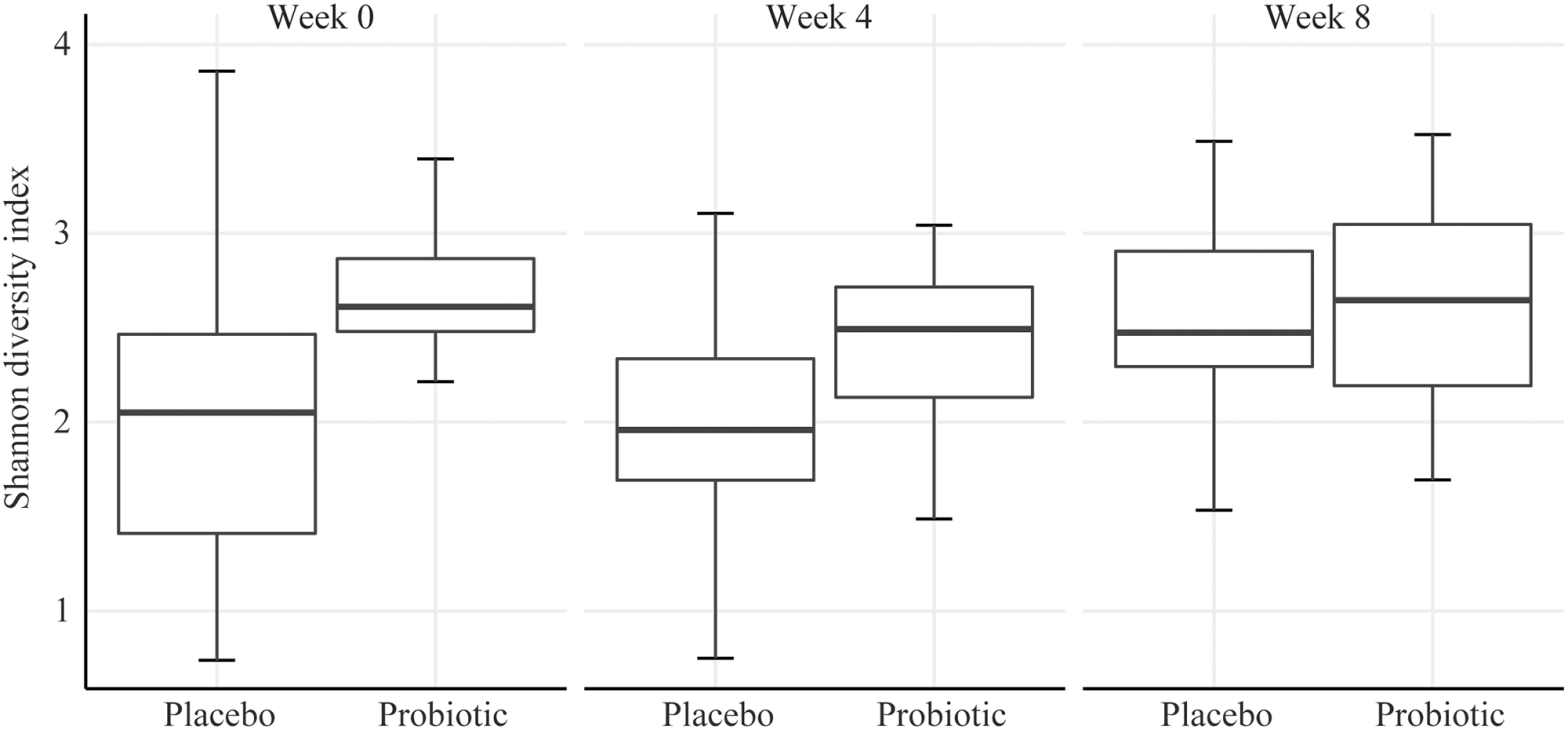
Microbial α-diversity as measured by the Shannon diversity index. The Shannon diversity index, which is a heterogeneity measure that combines richness and evenness components of microbial diversity does not significantly differ when subjects take probiotics.

We used the Jaccard similarity coefficient to consider differences in taxonomic presence or absence between treatment and across time points. The same treatment (*p* = 0.001) and time effects (*p* = 0.001) were identified; however, pairwise comparison and FDR correction again did not reveal significant comparisons that were meaningful. The α-diversity, as measured by the Shannon diversity index, revealed a significant treatment effect (*p* = 0.034) (Fig 1). However, after pairwise comparison and FDR correction, no comparisons were significant.

**Fig 2:**
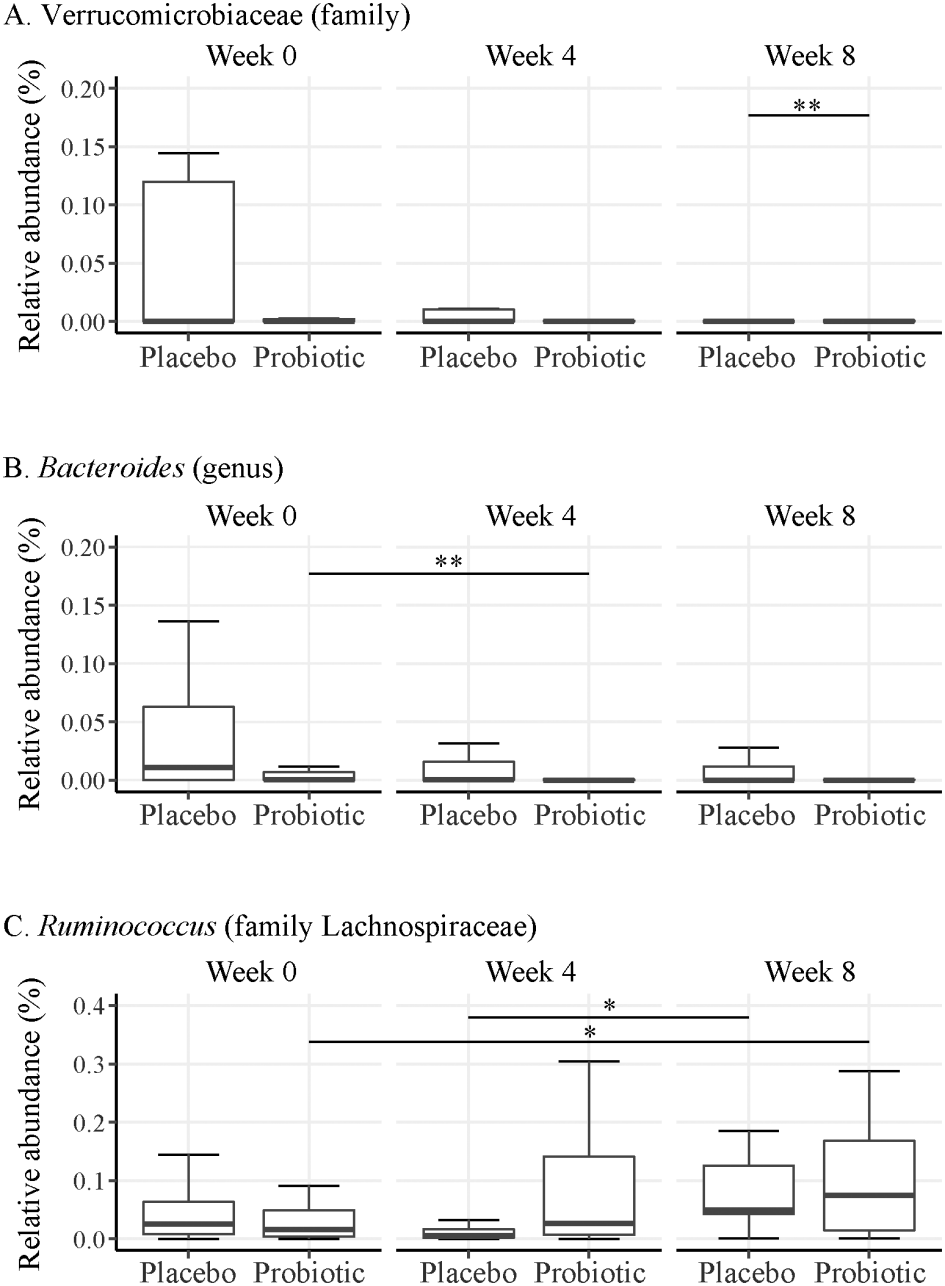
Taxa were found to be differentially abundant depending on treatment and time point. (A) Subjects in the probiotic group had a significantly lower abundance of the bacterial family Verrucomicrobiaceae at week 8 than placebo treated subjects; (B) Members of the bacterial genus *Bacteroides* were significantly reduced in abundance between weeks 0 to 4 in probiotic treated subjects; (C) *Ruminococcus* (family Lachnospiraceae) tended to be more abundant at week 8 than week 4 within the placebo group and at week 8 than week 0 within the probiotic group; * 0.05 < *p* < 0.1 and **p ≤ 0.05.

When examining taxonomic differences, several taxa differed significantly across time or treatment; however, none differed significantly for the time:treatment interaction. For taxa significant prior to FDR correction, we examined pairwise comparisons for significant treatment differences within time point and for significant time point differences within treatment. After FDR correction, we found that the family Verrucomicrobiaceae was significantly lower in the probiotic group compared to placebo at week 8 (*p* = 0.036) and the genus *Bacteroides* significantly decreased from week 0 to week 4 in the probiotic group (*p* = 0.046) (Fig 2A and 2B). We also found that the genus *Ruminococcus* (family Lachnospiraceae) tended to be higher in both the probiotic (*p* = 0.065) and placebo (*p* = 0.092) groups at week 8 relative to previous weeks (Fig 2C).

**Fig 3:**
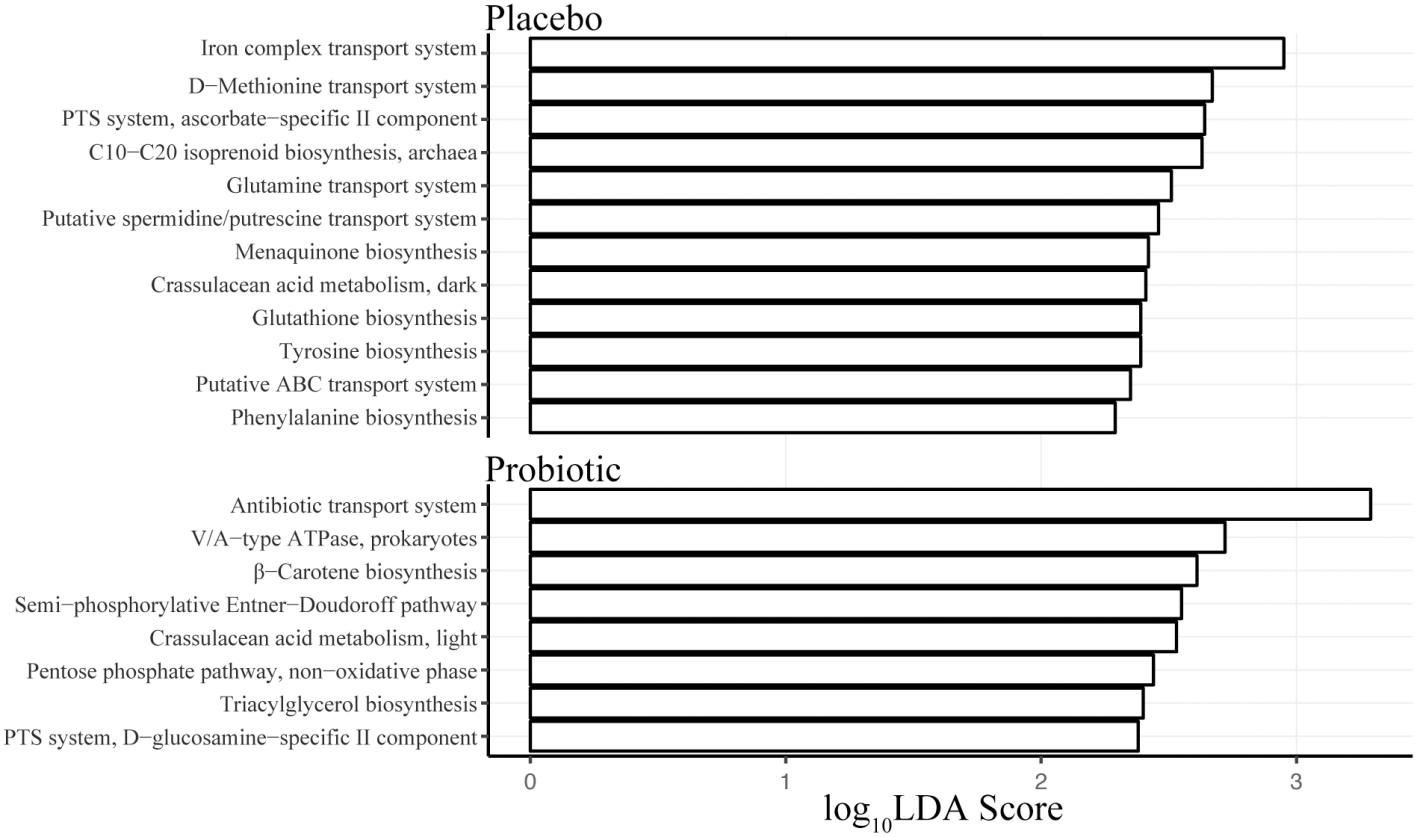
Predicted functional profiling of the GI microbiome in placebo or probiotic treated subjects undergoing CDI. Profiling of KEGG modules was based on 16S rRNA marker gene sequences from week 4 using PICRUSt. The α parameter for pairwise tests was set to 0.05 for class normality and the threshold on the logarithmic score of LDA analysis was set to 2.0. Modules that differed significantly in abundance between the treatment groups are displayed with the respective LDA score.

Given the observed taxonomic differences between the probiotic and placebo groups at week 4 and that this is the point at which treatment ended, we further assessed this time point for predicted functional pathways based on community structure. Our functional mapping of the 16S rRNA data using PICRUSt showed pathways and structural complexes that were the major modules enriched in either the placebo or probiotic treatment groups at week 4 (Fig 3). In the placebo group, enriched modules largely included biosynthesis pathways for amino acids like tyrosine (M00040) or phenylalanine (M00024) and cofactors like vitamin K (M00116). Additionally, enriched modules included structural complexes for amino acid transport, such as for glutamine (M00227) or methionine (M00238), and for substrates necessary in bacterial metabolism (M00283). The module with the highest LDA score in the placebo group was the iron complex transport system (M00248). The significantly abundant modules predicted for the probiotic group included those for carbon (M00308 or M00007) or glycerolipid (M00089) metabolism. The module with the highest LDA score was a structural complex for the transport of antibiotics (M00248).

## Discussion

Clinical studies have found that probiotics administered during antibiotic treatment may be effective for primary prevention of CDI [27]. In the PICO study, our group found that subjects administered a multi-strain probiotic alongside antibiotics had significantly improved diarrheal outcomes compared to subjects administered a placebo control [12]. In the present study, we used stool samples collected from subjects enrolled in PICO to identify the structural changes to the GI microbiota in subjects taking probiotics.

We found that despite significant main effects, there was neither a meaningful time nor treatment effect on the overall microbiome composition within pairwise comparisions among the study groups. Additionally, α-diversity, as measured by the Shannon index, indicated neither treatment nor time impacted the overall richness and evenness of the microbial communities among the study groups. These data suggest the multi-strain probiotic at the tested dose lacked the ability to significantly impact diversity of the microbiota in the current study. In a recent systematic review, it has been suggested that probiotics have no effect on the fecal microbiota composition when compared to placebo [28,29]. Many variables such as probiotic dose, strain, and patient demographics across studies make it difficult to draw conclusions on probiotic efficacy. In the present study, it is possible that the probiotic dose presents an intrinsic quantitative gap when considering its ability to alter the enormous quantity of native microorganisms already residing in the GI tract [30]. Further, the ability for probiotics to colonize the gut lumen, particularly if they are not adapted to the human gut, is likely strain specific [31,32]. As the multi-strain probiotic employed in this study largely contains strains of dairy origin, their impact on fecal microbiota diversity, if such an impact exists, may be difficult to identify.

Despite the lack of effect on community diversity, we sought to identify specific taxonomic changes among the treatments and time points in this study. First, we identified the family Verrucomicrobiaceae as significantly lower in the probiotic treatment group at week 8 compared to the placebo group. Interestingly, the presence or absence of Verrucomicrobiaceae has previously been associated with susceptibility for CDI. It was found that the fecal microbiota of mice treated with the broad spectrum antibiotic tigecycline to induce CDI susceptibility had an increased abundance of Verrucomicrobiaceae and Enterobacteriaceae than saline treated controls [33]. These taxonomic alterations, which coincided with severe CDI, eventually returned to baseline levels in mock-infected mice after cessation of tigecycline treatment. Though the increase in Verrucomicrobiaceae in the tigecycline study is presumably due to specificity of the antibiotic, our results suggest that probiotic treatment may limit Verrucomicrobiaceae proliferation for up to 4 weeks after treatment, which could reduce the risk for recurrent CDI and allowing for our previously observed reduction in the number of diarrhea days experienced for CDI subjects. Similar alterations to Verrucomicrobiaceae abundance were observed in a clinical study with subjects experiencing recurrent CDI who underwent a fecal microbiota transplantation (FMT) [34]. Recipients of the FMT in this study rapidly obtained a normalized microbiota structure and metabolic composition which coincided with clinical recovery. Taxonomic alterations associated with recovery included a significant reduction in the abundance of Enterobacteriaceae, Veillonellaceae, and Verrucomicrobiaceae and the proliferation of bile salt hydrolyzing microbiota. The authors concluded that restoration of a community composition which can support bile acid metabolism can limit *C*. *difficile* spore germination and allow for clinical recovery of subjects undergoing CDI.

We found the bacterial genus *Bacteroides* as significantly decreased in the probiotic group from week 0 to week 4. Previous studies suggest that reductions of *Bacteroides* are common with antibiotic treatment [35]. However, it is also known that the probiotic mixture used in our study may stabilize the antibiotic-associated disturbances to *Bacteroides* abundance [35]. Antibiotic disturbances in combination with the known antagonistic activity of *C*. *difficile* against *Bacteroides* suggests the probiotic treated subjects in our study are still undergoing an active CDI at week 4 [36]. Additionally, these results suggest that though we previously observed reduced symptomatic outcomes in probiotic treated subjects, the effects of CDI cannot be completely overcome by probiotic supplementation alone.

Other compositional changes were observed in members of the bacterial family Lachnospiraceae. Specifically, the genus *Ruminococcus* was identified as trending toward increased abundance within both treatment groups over time. Within the placebo group, *Ruminococcus* increased between week 4 and week 8 while the probiotic group increased between week 0 and week 8. Although the trend is not confined to the probiotic treatment group, it is possible that the increased abundance of members within Lachnospiraceae still play a role in the improved diarrheal outcomes observed in the PICO study. In a prospective study of CDI patients, *Ruminococcus* was identified as part of a microbiota signature that was associated with intact colonization resistance and suppression of *C*. *difficile* [37]. The authors noted that *Ruminococcus* is known to produce the lantibiotic Ruminococcin A in the presence of trypsin, which inhibits the *in vitro* growth of *C*. *difficile* [38]. In accordance with this study others have identified *Ruminococcus* as a native member of the microbiota that is differentially depleted during CDI [39]. In the current study, we would expect that by week 8 the microbiota of subjects in both groups of the current study would begin to approach its native structure and could explain why both treatment groups at week 8 would have an abundance of *Ruminococcus* that tends to be higher relative to prior time points. A study examining the same multi-strain probiotic indicated that *Ruminococcus* is significantly represented during probiotic treatment and that it is a taxa that is significantly more abundant in a stable microbiota [35]. Lastly, as a proof-of-concept several *Ruminococcus* isolates have been included in a synthetic stool mixture that was shown to be an effective therapy for treating recurrent CDI by colonic infusion [40]. Though in the current study we only identified a trend in *Ruminococcus* increases, determining whether the probiotic used in the present study can assist in restructuring the microbiota to increase levels of *Ruminococcus* would be beneficial to future *C*. *difficile* research with probiotics.

Although we observed taxonomic differences in GI microbiota composition between placebo and probiotic groups, it has been demonstrated that probiotics do not need to change the composition of the bacterial community to alter community function [41]. To indirectly determine microbiome function in probiotic treated subjects, we computationally predicted the most relevant differences between the treatment groups upon cessation of treatment. We found subjects in the placebo group were significantly enriched in the iron complex transport system (M00240) at week 4. This prediction is in accordance with the prerequisite for iron acquisition during infection by *C*. *difficile* [42]. It is possible subjects in the placebo group undergo homeostatic mechanisms, such as iron sequestration by lactoferrin, to make iron unavailable to *C*. *difficile* in the GI tract. This would generate an iron sparse environment and a subsequent need for iron transport systems in members of the microbiome for which iron is an essential element.

Subjects from the probiotic treatment group were significantly enriched in the antibiotic transport system (M00248) at week 4. These transporter systems include ATP-binding cassette transporters that couple the transport of antibiotic through the cell membrane to ATP hydrolysis [43]. It is possible that commensal members of the GI microbiome in subjects administered probiotics may enhance their antibiotic efflux capabilities to better survive antibiotic treatment [44]. This mechanism, whether influenced by the probiotic strains or the observed taxonomic changes, is unknown in the present study. However, it may be a sign of reduced commensal sensitivity during antibiotic treatment, resulting in the improved diarrheal outcomes previously observed in the probiotic treated subjects.

The scope of this study was to identify the structural changes to the GI microbiome associated with supplemental probiotic treatment in subjects administered antibiotics for a primary episode of CDI. We identified taxa, namely Verrucomicrobiaceae, *Bacteroides*, and *Ruminococcus* as potentially involved in the improved diarrheal outcomes observed in subjects of the PICO study [12]. We also predicted functions associated with the microbiome of placebo and probiotic treated subjects that can serve as insight into future research questions.

However, the present study has limitations. First, although we collected serial stool samples, a longer follow-up period with collection of additional stool samples would have been useful for evaluating differences over time between treatment groups. Second, although we collected clinical and demographic data on participants, the small sample size did not permit multivariable analyses to adjust for confounding variables such as diet, comorbidities, and medications [45,46]. This is particularly relevant for *C*. *difficile*, as the available antibiotic treatments vancomycin, metronidazole, and fidaxomicin are known to impair the microbiota to different degrees [47,48]. Lastly, the use of predictive software to assign functional differences between treatment groups has limitations. These types of analyses are a cost-effective method for hypothesis generation as they have been shown to have high accuracy [24]. However, gene family predictions are based on taxonomic identification of microorganisms within a community, which is largely dependent on availability of genomic data for the taxa [24]. As such, metagenomic or metatranscriptomic methods would provide a much higher resolution of microbial communities. For probiotics to be considered as a possible adjunctive CDI therapy, understanding the associated impacts at the community level of the GI microbiota is important. Future studies should seek to examine the multi-strain probiotics in models where mechanistic investigations are possible.

## Supporting information

S1 Table Comparison of overall microbial community dissimilarities. (DOCX)
