# Supplementary material for: Oral probiotic combination of Lactobacillus and Bifidobacterium alters the gastrointestinal microbiota during antibiotic treatment for Clostridium difficile infection
Source: PLoS One. 2018 Sep 28;13(9):e0204253. doi: 10.1371/journal.pone.0204253 (PMC6161886; doi:10.1371/journal.pone.0204253)
Supplement: S1 Table — (DOCX) [file pone.0204253.s001.docx]

**S1 Table.** Comparison of overall microbial community dissimilarities

| Variable | Comparison | Bray-Curtis  FDR *p*-value |  | Jaccard  FDR *p*-value |
| --- | --- | --- | --- | --- |
|  | Week 0 vs. 4 | 0.317 |  | 0.324 |
| Placebo | Week 0 vs. 8 | 0.096 |  | 0.093 |
|  | Week 4 vs. 8 | 0.144 |  | 0.162 |
|  |  |  |  |  |
|  | Week 0 vs. 4 | 0.144 |  | 0.167 |
| Probiotic | Week 0 vs. 8 | 0.096 |  | 0.093 |
|  | Week 4 vs. 8 | 0.534 |  | 0.554 |
|  |  |  |  |  |
| Week 0 | Placebo vs. Probiotic | 0.705 |  | 0.672 |
| Week 4 | Placebo vs. Probiotic | 0.673 |  | 0.612 |
| Week 8 | Placebo vs. Probiotic | 0.916 |  | 0.952 |
